# Special Issue: Soft Photonic Crystals and Metamaterials

**DOI:** 10.3390/ma15228096

**Published:** 2022-11-16

**Authors:** Ivan V. Timofeev, Wei Lee

**Affiliations:** 1 Kirensky Institute of Physics, Federal Research Center KSC SB RAS, 660036 Krasnoyarsk, Russia; 2 Siberian Federal University, 660041 Krasnoyarsk, Russia; 3 Institute of Imaging and Biomedical Photonics, College of Photonics, National Yang Ming Chiao Tung University, Guiren District, Tainan 711010, Taiwan

Soft matters include polymers, liquid crystals, colloids, biological tissues, and many smart materials. Weak molecular interactions make them sensitive to tiny mechanical, thermal, electric, optical, and other external stimuli. Photonic applications often require soft materials with significant or, in some cases, extreme optical dispersion and non-linear and anisotropic properties. The optical response can be structurally amplified in two fairly distinct scales that are wavelength-comparable periods of soft photonic crystals and subwavelength-graded soft photonic metamaterials.

The reader is invited to explore the findings of interdisciplinary materials science research included in this Special Issue, including fresh concepts from physics, chemistry, biology, and technology. This Special Issue presents ten original open-access articles, of which three are editor’s choice articles. The collection can be divided into four categories. There are two papers related to smart windows (Tseng et al. [1] as well as Yokota et al. [2]). Two papers involve microparticle arrangement in colloidal and photonic crystalline structures (Seki et al. [3] and Alvarez et al. [4]). There are three related to switchable or tunable liquid crystal devices (Nys et al. [5], Yu et al. [6], and Bikbaev et al. [7]). The latter exploits light localization between the grating and the photonic crystal. Predicted by Tamm in 1932, this localized spot of light is called a Tamm plasmon, as it is a photon analogy of the electron state localized near the surface of a solid crystal. Finally, there are three more articles focusing on Tamm plasmons (Reshetnyak et al. [8], Avdeeva et al. [9], and Lin et al. [10]).

Tseng and coworkers [1] present a novel smart window utilizing salt and photochromic dichroic dye-doped cholesteric liquid crystal, that is essentially a soft photonic crystal. The smart window provides adjustable sunlight intensity and protects the privacy of people in a building. The functionality is based on the combination of two features: the darkening of a photosensitive layer upon exposure to ultraviolet light, and the response of cholesteric to voltage pulses. By independently switching both functionalities, four stable optical states are obtained: transparent, scattering, dark-clear, and dark-opaque. The presented work brings an interesting set of experimental data, including voltage, spectral, and temporal dependences, along with colored photos of the achieved optical states. The smart window offers passive automatic dimming and active haze control for privacy protection, exhibiting significant potential for applications.

The second paper on smart windows is contributed by Yokota et al. [2]. Fréedericksz transition in liquid crystals is one of the most classic and important concepts with real applications for liquid crystals in the society. Optical Fréedericksz transition is free of electrodes or wires and potentially more robust, however, it is not so popular because of the high threshold for light intensity. The paper strives to reduce the threshold until sunlight showing the dye doping has a great impact on the optical Fréedericksz transition, and that the host liquid crystal type also plays an important role. This is explained by the changes in the optical-electric torque, the elastic torque, and the dye torque. The threshold light intensity in trifluorinated liquid crystals is shown to be 42% lower than that in liquid crystals without fluorine substituents.

Colloidal and photonic crystalline structures are often fabricated by the arrangement of monodisperse microparticles. Seki and colleagues [3] report a one-pot hydrothermal synthesis of monodisperse magnetite microparticles. By adjusting the concentration of the reaction solution, the microparticle’s diameter is manipulated from 100 to 200 nm. Under an external magnetic field the microparticles in aqueous suspension form a periodic structure that possess Bragg reflection colors observed in the spectral range from 730 nm to 570 nm by increasing the magnetic field.

The paper by Alvarez et al. comes from an international research group combining seven members from Austria, Canada, and Taiwan [4]. It presents opalescent photonic crystals obtained via self-assembly of silica microparticles using a solvent evaporation method. Silica microparticles are synthesized by the sol-gel method. The microparticle diameter is precisely controlled by only changing the amount of the solvent used. Thorough characterization of quasi-hexagonal spatial order is presented in Appendix using Voronoi tessellations, pair correlation functions, and bond order analysis. In both Seki et al. [3] and Alvarez et al. [4], the Bragg functionality of microparticle arrays is suggested for full-color reflective displays, sensing materials, solar cells, and coatings.

Switchable diffraction gratings and patterns are a promising class of soft-matter metasurfaces for modulated electro-optic devices. Nys et al. [5] use a thin liquid crystal layer with periodic photoalignment at the surfaces to fabricate stable two-dimensional hexagonal diffraction patterns. The research is brilliant both in experiment and simulation. First, the patterns between a strong scattering state and a near clear state on linear variation in the photoalignment direction are studied by means of polarization optical microscopy and light diffraction. Then, the numerically restored director configuration reveals a 3D superstructure, where all the twist conflicts in the confining substrates produce out-of-plane director reorientation without disclinations. The three-fold and six-fold twist angles yield hexagonal ordering that can be reversibly and continuously switched by small voltage.

Yu et al. [6] have investigated the uniform lying helix texture in a polymer-network cholesteric liquid crystal layer composed of a binary achiral mixture (of E7 and the bent-core dimer CB7CB). This 5 μm thick soft-matter film has fascinatedly tunable helicity with its helical axis along the substrate plane and its pitch of around 100 nm, which is shorter than the wavelengths of visible light. Applied voltage can generate two types of helix reorientation. At low voltage, the flexoelectric effect produces periodic splay-bend deformation of in-plain optical axis. Beyond the threshold voltage the dielectric effect occurs and the helix is gradually unwound until transformed to the helix-free homeotropic state. Polymer networks are used to stabilize this state against other orientations with lower free energy, such as planar, focal conic, and 3D-helical blue-phase states. A bifunctional monomer and a trifunctional monomer are utilized to demonstrate the advantage of photo-copolymerization for a temperature-stable cell. The final cell optical properties are guaranteed to be reversibly controlled by both voltage frequency and amplitude. A detail account of the novel incorporation of the liquid crystal dimer CB7CB is clearly stated in the paper.

Bikbaev et al. [7] are engaged in a challenge for mirrorless beam steering by generalized diffraction in voltage-tunable metagratings. With this purpose in mind, they consider a subwavelength grating patterned on top of a Bragg reflector. Under the metagrating strips the electromagnetic field can be efficiently localized in above-mentioned Tamm plasmon. This narrow resonance is extremely sensitive to refractive index of a top layer, which makes it possible to control each strip radiation phase in a wide range. This effect can be realized by the top layer made of transparent conductive material, such as indium tin oxide. The bias voltage up to 5 V leads to an increase in the volume concentration of the charge carriers in few nanometer vicinity of the layer boundary. As a result, the real part of the complex dielectric permittivity becomes negative, acquires metallic properties, and shifts the scattering phase by more than 200 degrees. Changing the number of strips with different applied bias voltage allows for efficient switching in first-order diffraction. In addition, it is shown that the reflected beam can be controlled by a continuous phase change along the metagrating. Definitely, this method increases the beam steering angular resolution.

Reshetnyak et al. [8] focus on the above-mentioned Tamm plasmons for the case of a thin silver layer adjacent to a rugate filter—a dielectric thin film with a smooth periodic profile of the refractive index, acting like a Bragg mirror. The smoothness helps to suppress sidelobes outside of a photonic band gap and provides several extra degrees of freedom for optimizing optical characteristics compared to multilayer Bragg mirrors. Surprisingly, the harmonic refractive index profile allows for convenient analytical expressions in regard to Tamm plasmon transmission and reflection peaks and dips. The analytics shows dependence on refractive index contrast, the metal layer thickness, and the external medium refractive index. As an example, it is shown that the Tamm plasmon wavelength can be at any point within the photonic band gap determined by the refractive index profile. The analytical approach complies quite well with exact numerical simulations in a broad range of experimentally available parameters.

Avdeeva et al. [9] investigate chiral Tamm plasmons—a class of the aforementioned Tamm plasmons with the broken mirror symmetry. The chirality of state originates from cholesteric liquid crystal, a soft self-organizing photonic crystal. A crucial requirement for such a state emergence is the presence of a polarization-preserving anisotropic mirror instead of conventional metallic layer. This mirror is not necessarily chiral, and therefore preserves the handedness of reflected light polarization compared to incoming one. Extensive dye-doping makes cholesteric highly dispersive material leading to spectral splitting of resonant features, such as the edge and defect modes of photonic crystal. The paper proves that chiral Tamm state spectral peak can be resonantly split as well, which is important for tunable chiral microlasers. The splitting is most pronounced when dye resonance frequency coincides with the frequency of the Tamm state. In this case, the reflectance, transmittance, and absorptance spectra show two distinct Tamm modes. For both modes, the field localization is at the interface of the polarization-preserving anisotropic mirror and the cholesteric.

Chiral Tamm plasmon was for a long time considered as a fantastic theoretical monster because of serious experimental restrictions imposed by the polarization-preserving anisotropic mirror. Lin et al. [10] proudly win the challenge of in-principle experimental realization of this chiral monster. They adopt a recently suggested 200 nm thick reflective half-wave phase plate implemented as a golden metasurface. Simultaneously they use an extremely birefringent stable cholesteric liquid crystal (Δ*n* = 0.33). The 3 μm thick cholesteric layer provides almost optimal coupling of chiral Tamm plasmon when reflectance falls down to 20%. Moreover, the suggested design renders both phase and polarization matching. Furthermore, by temperature-changing the center wavelength of the cholesteric band gap with different pitches, the resonance wavelength of Tamm plasmons is tuned flexibly. This phenomenon is essential for advancing technical applications in novel types of optical switches, biosensors, polariton microlasers, and mirrorless lidars.

We would like to thank all the international teams of creative intellectuals and specialists who have made this Special Issue possible. First are the authors who have kindly prepared their contributed or invited papers. In addition, we would like to thank all the peer reviewers who participated in this Special Issue for their invaluable time to offer professional and constructive comments, which definitely helped promote the quality of the scientific content of each published paper. Our gratitude is extended to the people we interacted with at MDPI for their hard work and considerate coordination throughout the entire editorial process.
